# Icariin improves oxidative stress injury during ischemic stroke via inhibiting mPTP opening

**DOI:** 10.1186/s10020-024-00847-2

**Published:** 2024-06-05

**Authors:** Zhiyong Zhou, Weili Li, Lu Ni, Tianlun Wang, Yan Huang, Yuanqi Yu, Mingxin Hu, Yinling Liu, Jin’e Wang, Xiaofei Huang, Yanyan Wang

**Affiliations:** 1 Third-grade Pharmacological Laboratory on Traditional Chinese Medicine Approved by State Administration of Traditional Chinese Medicine, Yichang, 443002 P. R. China; 2 College of Medicine and Health Sciences, China Three Gorges University, Yichang, 443002 P. R. China; 3 College of Basic Medical Sciences, China Three Gorges University, Yichang, 443002 P. R. China; 4 The First College of Clinical Medical Science, China Three Gorges University, Yichang, 443000 P. R. China

**Keywords:** Icariin, Cerebral ischemia-reperfusion, ROS, Mitochondrial permeability transition pore, Ischemic stroke

## Abstract

**Background:**

Ischemic stroke presents a significant threat to human health due to its high disability rate and mortality. Currently, the clinical treatment drug, rt-PA, has a narrow therapeutic window and carries a high risk of bleeding. There is an urgent need to find new effective therapeutic drugs for ischemic stroke. Icariin (ICA), a key ingredient in the traditional Chinese medicine Epimedium, undergoes metabolism in vivo to produce Icaritin (ICT). While ICA has been reported to inhibit neuronal apoptosis after cerebral ischemia-reperfusion (I/R), yet its underlying mechanism remains unclear.

**Methods:**

PC-12 cells were treated with 200 µM H_2_O_2_ for 8 h to establish a vitro model of oxidative damage. After administration of ICT, cell viability was detected by Thiazolyl blue tetrazolium Bromide (MTT) assay, reactive oxygen species (ROS) and apoptosis level, mPTP status and mitochondrial membrane potential (MMP) were detected by flow cytometry and immunofluorescence. Apoptosis and mitochondrial permeability transition pore (mPTP) related proteins were assessed by Western blotting. Middle cerebral artery occlusion (MCAO) model was used to establish I/R injury in vivo. After the treatment of ICA, the neurological function was scored by ZeaLonga socres; the infarct volume was observed by 2,3,5-Triphenyltetrazolium chloride (TTC) staining; HE and Nissl staining were used to detect the pathological state of the ischemic cortex; the expression changes of mPTP and apoptosis related proteins were detected by Western blotting.

**Results:**

In vitro: ICT effectively improved H_2_O_2_-induced oxidative injury through decreasing the ROS level, inhibiting mPTP opening and apoptosis. In addition, the protective effects of ICT were not enhanced when it was co-treated with mPTP inhibitor Cyclosporin A (CsA), but reversed when combined with mPTP activator Lonidamine (LND). In vivo: Rats after MCAO shown cortical infarct volume of 32–40%, severe neurological impairment, while mPTP opening and apoptosis were obviously increased. Those damage caused was improved by the administration of ICA and CsA.

**Conclusions:**

ICA improves cerebral ischemia-reperfusion injury by inhibiting mPTP opening, making it a potential candidate drug for the treatment of ischemic stroke.

**Graphical Abstract:**

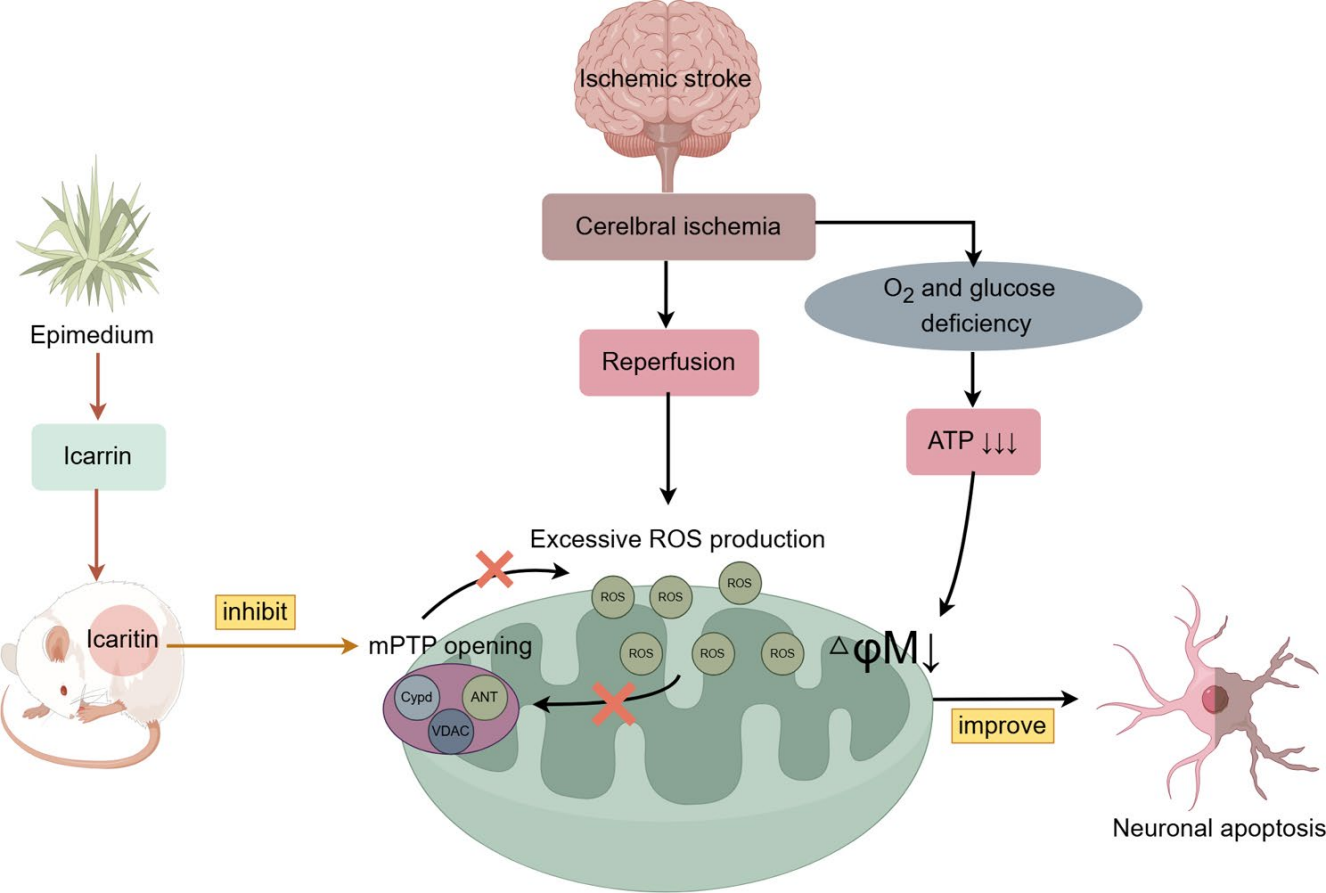

## Introduction

Ischemic stroke, a clinical syndrome resulted from neuronal ischemia, constitutes 80–85% of stroke and carries high rates of disability and mortality(Xie et al. 2020). According to the 2020 American Heart Association statistics, approximately 795,000 people experience a new or recurrent stroke each year (Virani et al. 2020). Hypertension, smoking, and unhealthy diet are common factors leading to stroke. Importantly, aging is an independent risk factor for stroke, and with the aging population, the risk of lifelong stroke is constantly increasing(Tsao et al. 2023).

Ischemic stroke damage occurs in two stages: cerebral ischemia and reperfusion. During the cerebral ischemia stage, due to restricted circulation, neuronal electrical function is lost, leading to neuronal damage and cell death. Reperfusion generates a large amount of reactive oxygen species (ROS) through various mechanisms, exacerbating damage and being the primary cause of I/R injury (Allen 2009; Ray et al. 2012). Under normal physiological conditions, intracellular ROS participate in cellular defense mechanisms to modulate cellular signaling, such as cell cycle, gene expression, cell survival and apoptosis (Groeger et al. 2009; Patten et al. 2010; Dasuri, et al. 2013), playing a crucial role in maintaining cellular homeostasis. However, during reperfusion injury, mitochondrial respiratory chain uncoupling occurs, accompanied by energy consumption (Turrens 2003). Superoxide anions are converted to H_2_O_2_ and its free radicals, leading to oxidative stress and uncontrolled ROS production (Saeed et al. 2007; Chen et al. 2018). Subsequently, excessive ROS causes neuronal damage through lipid peroxidation, DNA damage, and protein oxidation (Qu et al. 2016; Katsu et al. 2010; Yunoki, et al. 2014). Therefore, maintaining mitochondrial homeostasis and inhibiting excessive production of ROS are key targets for improving I/R injury.

Mitochondria serve as the major sub-cellular organelles for ROS production. The mPTP is a non-specific channel located in the inner mitochondrial membrane, composed of voltage-dependent anion channel (VDAC), adenine nucleotide translocase (ANT), and cyclophilin (CYPD) (Chaudhuri et al. 2016; Skemiene et al. 2020). Under normal conditions, transient and reversible opening of the mPTP can release accumulated ROS and maintain mitochondrial homeostasis. However, under pathological conditions of oxidative stress and elevated intracellular Ca^2+^, the mPTP continues to open, leading to excessive ROS release (Hausenloy et al. 2004; Zorov et al. 2014)and mitochondrial dysfunction (Chanoit et al. 2011; Neumar 2000) Previous studies have been demonstrated continuous mPTP opening in neuronal mitochondria during ischemic stroke (Kinnally et al. 2011; Nazareth et al. 1991; Li, et al. 2020). Pharmacological inhibition of mPTP overactivity effectively mitigates neuronal damage induced by ischemia-reperfusion (Liu et al. 2022; Zhou et al. 2017).

**Fig. 1:**
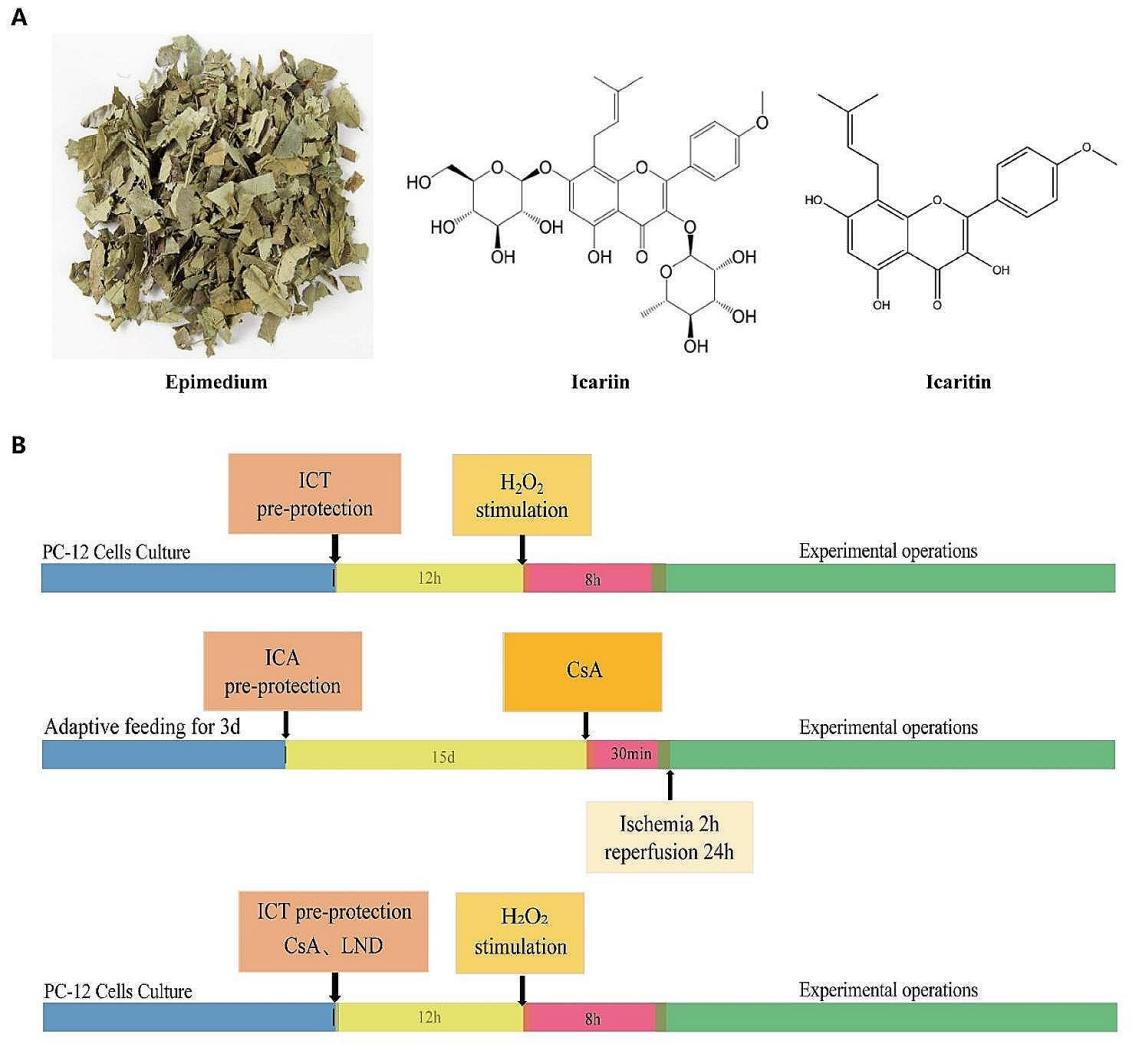
Image of Epimedium(left), chemical structure of Icariin (middle), and Icaritin (right) (**A**); Schematic diagram of the experimental protocol (**B**)

Icariin (ICA), the active compound in traditional Chinese medicine Epimedium, undergoes metabolism in vivo to produce Icaritin (ICT) (Fig. 1A), which exhibits diverse pharmacological effects, including antioxidant activity, neuroprotection, and immune regulation (Xu et al. 2021a; Song et al. 2016; Tao, et al. 2021). Studies have demonstrated that ICA can reduce ROS content (Li and Meng 2019; Zhu et al. 2010), and enhance mitochondrial function, protecting neurological function in a MCAO model(An et al. 2021). Moreover, ICT demonstrates superior neuroprotective effects compared to ICA in acute cerebral ischemic stroke models, reducing neurological damage, infarct volume, and histopathological changes (Wu et al. 2021, 2022). Furthermore, ICT inhibits H_2_O_2_-induced oxidative stress, preserves mitochondrial member potential, and maintains mitochondrial morphology (Fan et al. 2023). Nevertheless, the underling molecular mechanism remains incompletely elucidated.

To investigate the protective effect of ICA against I/R injury, we established an oxidative stress model in vitro and evaluated its primary active component, ICT, on cell viability and oxidative stress markers following I/R insult. Furthermore, to assess whether the protective mechanism of ICT involves mPTP, we employed calcein staining and immunoblotting techniques to monitor changes in mPTP levels. Additionally, to assess ICA’s impact on MCAO rats, we monitored neural function and neuronal morphology post-MCAO surgery, along with extracting brain proteins to detect alterations in relevant protein expressions. Lastly, to delve deeper into the role of mPTP in ICA’s anti-I/R injury effect, we utilized a combination of mPTP inhibitors (Cyclosporin A, CsA) and agonists (Lonidamine, LND) to investigate the combined effect on mPTP’s open state.

## Materials and methods

### Antibodies and reagents

ICA was purchased from Chengdu Phytolabel Chemical Pure Biotechnology Co. CsA ((≥ 98%, B28163)) and LND (99%, S80752) were purchased from Shanghai Yuanye Biotechnology Co. H_2_O_2_ was purchased from Shanghai State Pharmaceutical Group at a concentration of 9.908 mmol/L. The MMP detection kit (JC-1) and mPTP detection kit were purchased from Beyotime. The ROS detection kit (DCFH-DA), RIPA buffer and BCA protein assay kit were purchased from Applygen. The Annexin V-FITC/PI apoptosis detection kit was obtained from Jiangsu Keygen. The ECL chemiluminescence detection kit was purchased from Servicebio, and the PVDF membrane was purchased from Millipore. Anti-β-Actin (GB11001) was obtained from Servicebio, anti-VDAC (#83,506, #4661) from Cell Signaling, anti-Bcl2 (26593-1-AP), and anti-Bax (50599-2-Ig) from Proteintech, and anti-CypD (A3208) from ABclonal. All these reagents and compounds are standardized and commercially available. TTC was purchased from SIGMA Co.

### Cell culture

Highly differentiated PC-12 cells were purchased from the Cell Bank of the Chinese Academy of Sciences and cultured in 1640 medium containing 10% fetal bovine serum and 1% penicillin/streptomycin at 37 °C with 5% CO_2_.

### Cell viability

Cell viability was measured by MTT (M8180, Solarbio, Beijing, China). PC-12 cells (5 × 10^3^/well) were seeded in flat-bottomed 96-well plates and cultured for 24 h. Subsequently, 20 µL MTT solution was added to each well and incubated for 4 h. After removing the supernatant, 200 µL of DMSO was added to each well and mixed for 10 min. The absorbance of each well was measured at 570 nm using a microplate reader, and cell viability was calculated using the formula: Cell viability = (OD of experimental group-OD of blank group) / (OD of control group-OD of blank group).

### Measurement of intracellular ROS levels by Flow Cytometry

PC-12 cells (1 × 10^5^/well) were seeded in 6-well plates and cultured for 24 h. Following cell digestion, PBS wash, and centrifugation, the DCFH-DA probe (diluted 1:10000 in serum-free1640 medium) was added to resuspend the cell pellets (200 µL probe per well). The cells were then incubated at 37 °C for 30 min in the dark. Afterward, the samples were washed with PBS and analyzed by flow cytometry or an inverted fluorescence microscope.

### Determination of apoptosis

Following collection, cells were washed with PBS. Afterward, 300 µL of binding buffer was added to resuspend the cells, followed by the addition of 3 µL each of Annexin V-FITC and PI dye. After thorough mixing, the mixture was incubated in the dark at room temperature for 15 min and immediately analyzed by flow cytometry.

### Assessment of mPTP status

As per the kit instructions, 200 µL of Calcein AM staining solution and fluorescence quenching working solution were added to each well upon the completion of cell growth. After incubating for 40 min at 37 °C, the staining solution was removed, and the preheated medium was added for an additional 30 min incubation before washing with PBS. Subsequently, 4% paraformaldehyde was added for fixation, incubating for 40 min, following by PBS washing. Finally, samples were mounted with an anti-fluorescence quencher and observed using a laser confocal microscope.

### Detection of the mitochondrial membrane potential by JC-1 staining

The JC-1 staining working solution was prepared according to the instructions. After cell culture is completed, 200 µL of culture medium and an equal volume of JC-1 staining working solution were added to each well of 6-well plates followed by incubation in the dark at 37 °C for 30 min. Afterward, the supernatant was aspirated and cells were washed gently with JC-1 staining buffer. Finally, 500 µL of cell culture medium was added and cells were observed under an inverted fluorescence microscope.

### Animals and treatments

The male Sprague Dawley rats at 160 ~ 180 g of body weight were purchased and fed by the Animal Center of China Three Gorges University, Yichang, China. (Certificate No.: SYKX (E) 2022-0061). Rats were housed in an environmentally controlled facility (12:12 light/ dark cycle; 22–25℃; food and water ad libitum).

Animals were randomly assigned to five groups, as outlined in Fig. 1B:

1. MCAO: Rats underwent the MCAO procedures as described in previous studies (Ma et al. 2020). Briefly, after isoflurane anesthesia, the right common carotid artery (CCA) and external carotid artery (ECA) of the rats were ligated, and a rubber-coated nylon filament (Shenzhen Reward Life Technology Co., LTD., China) was slowly inserted through the incision on the CCA until slight resistance was felt, indicating blockage of the middle cerebral artery (MCA). After 2 h, the filament was withdrawn to allow reperfusion for 24 h.
2. Sham: Rats underwent the same surgical procedure as in the MCAO groups, but without insertion of the filament into the MCA. Rats received 5% CMC-Na solution (i.g, at 9 a.m. and 9 p.m.) for 15 days.
3. MCAO + 10 mg/kg ICA: Rats received ICA (10 mg/kg, i.g, at 9 a.m. and 9 p.m.) for 15 days before MCAO. ICA was dissolved in 5% CMC-Na solution.
4. MCAO + 30 mg/kg ICA: Rats received ICA (30 mg/kg, i.g, at 9 a.m. and 9 p.m.) for 15 days before MCAO.
5. MCAO + 15 mg/kg CsA: Rats received CsA (15 mg/kg, i.p) 30 min before MCAO.

### Neurological deficits assessment

Neural impairment in rats was evaluated using ZeaLonga’s methods(Longa et al. 1989), with scores ranging from 0 to 4 points: 0 points indicating no obvious nerve damage, 1 point indicating incomplete extension of the front paw, 2 points indicating circling to the opposite side, 3 points indicating falling to the left or no autonomous movement, 4 points indicating involuntary walking. Evaluations were performed 24 h after MCAO.

### TTC staining

As previously stated, rats underwent treatment with various formations. Subsequently, after 24 h, the brains of the rats were initially chilled at -20℃ for 30 min. Promptly following this, the coronal plane was swiftly sliced into five sections, each measuring 2 mm in width, proceeding from the anterior to the posterior region.

After 24 h of reperfusion injury, collect the rat brain tissue and freeze at -20 ℃ for 30 min. The coronal plane was swiftly sliced into five sections, from the anterior to the posterior region, each measuring 2 mm in width.

These sections were subsequently stained with 0.5% TTC in PBS solution (Sigma Chemical Co.) at 37 °C for 30 min, with rotation every 10 min. Following staining, the stained section was fixed with paraformaldehyde for 24 h and photographed. Only the second slice adjacent to the olfactory bulb was utilized for analysis and imaging. The calculation of single infarct volume was determined by multiplying the infarct size per slice by the average section thickness (2 mm). Image J was used for analysis to calculate the infarct volume of each brain.

### Hematoxylin & eosin (H&E) staining

After 24 h of reperfusion, the rats were anesthetized with 10% chloral hydrate. Then, they were perfused through the heart with 0.9% normal saline, followed by 4% paraformaldehyde. After that, the brain tissues were taken out for gradient dehydration and embedding. They were then cut into 4 μm-thick slices, which were baked at 60 °C for 5 h. The slices were stained with hematoxylin and eosin and sealed with neutral glue. Randomly selected three rats from each group were used for wax block preparation, and 3 non-adjacent slices were chosen for microscopic observation of staining intensity. After randomly selecting 10 fields (40×) from each slice for observation.

The histo-morphological evaluation was carried out in a double-blind manner by a pathologist who was unaware of the experiment design. The pyramidal cells in the slices with large and round nuclei, light and clear staining, obvious nucleoli and nuclear membranes, and abundant cytoplasm were identified as normal cells. Those with dark staining, blurred and atrophic nuclear membranes were identified as pathologically degenerative cells.

### Nissl staining

Briefly, three rats were randomly selected from each group, and three non-adjacent slices were chosen from their wax blocks. After dewaxing with xylene and graded alcohol, the slices were placed in double-distilled water for 5 min. They were then incubated with toluidine blue solution at 37 °C for 5 min, followed by washing with distilled water 3–5 times. After drying, the slices were placed in xylene for 5 min and then sealed with neutral resin. Staining intensity was observed under a microscope, and 10 random fields (40×) were selected from each slice for qualitative analysis.

The histo-morphological evaluation was carried out in a double-blind manner by a pathologist who was unaware of the experiment design. Nissl bodies that are atrophied and exhibit a sieve-like pattern were identified as degenerative cells.

### Western blot

The ischemic penumbra cortex was harvested and homogenized on ice with lysis buffer; the homogenate was centrifuged to obtain the supernatant. Then quantified the protein following the BCA kits protocols (P1513-1, APPLYGEN).

Equal amounts of protein (30 µg in 10 µL) from total lysates were electrophoresed on 8% or 12% SDS-PAGE gels and transferred to PVDF membranes. After blocking with 5% non-fat milk at room temperature for 1 h, membranes were incubated with primary antibody at 4 °C overnight. Following washing in TBST buffer for 3 times, membranes were incubated with secondary antibody at room temperature for 1 h. Digital images of the membranes were captured by the chemiluminescence method using an ECL kit (Servicebio, Wuhan, China). Protein levels in samples were quantified by Image J 6.0 software.

### Statistical analysis

Statistical analyses were performed using GraphPad Prism version 8.0. The Shapiro-Wilk test confirmed that the quantitative data followed a normal distribution. The data were represented as mean ± SD. To assess the significance of differences between two groups, an Independent-Samples T-test was conducted. For multiple comparisons, a one-way ANOVA followed by Turkey’s post hoc analysis was utilized. A p-value below 0.05 was deemed statistically significant.

### Result

#### Establishment of H_2_O_2_ induced oxidative damage model

**Fig. 2:**
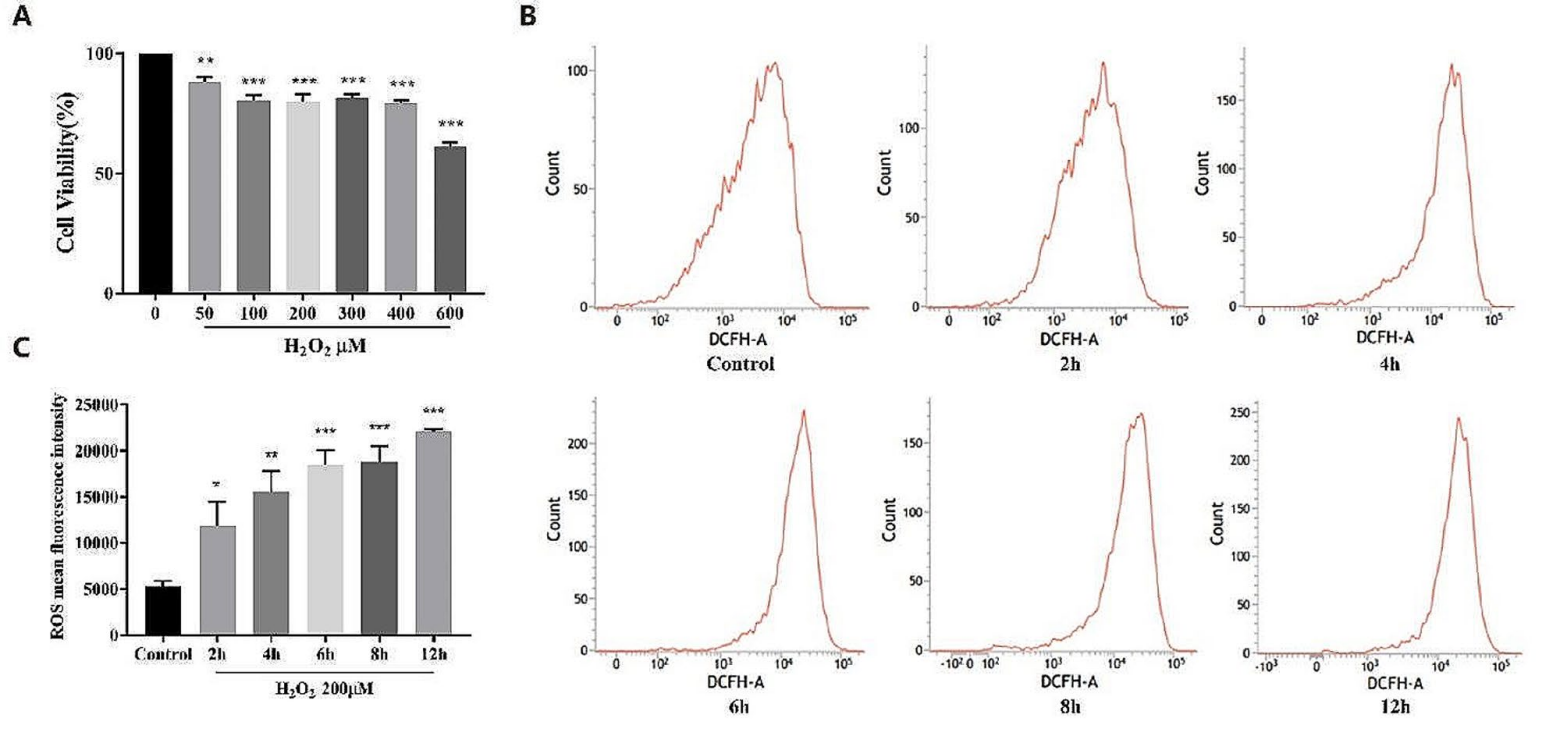
Establishment of the H_2_O_2_ induced oxidative damage cell model. After stimulating the PC-12 cells by different concentrations of H_2_O_2_ for 8 h, cell viability was measured by the MTT assay (**A**). Cells were exposed to 200µM H_2_O_2_ for 2, 4, 6, 8 and 12 h, and ROS levels were detected by the flow cytometry (**B**-**C**). (Data were represented as mean ± SD, *n* = 3, **P < 0.01 vs. control, ***P < 0.001 vs. control)

H_2_O_2_ is commonly used to induce oxidative damage, mimicking in vitro I/R models. To screen out the optimal stimulation condition, we assessed cell viability through the MTT method. As shown in Fig. 2A, at a concentration of 100–400 µM H_2_O_2_, the cell survival rate was around 80%. We chose 200 µ M conducts subsequent testing. Meanwhile, flow cytometry results revealed a time-dependent increase in intracellular ROS levels with prolonged H_2_O_2_ exposure (Fig. 2B-C). At 8 h, the ROS level increased to three times the normal control. Based on this, 200 µM H_2_O_2_ stimulation for 8 h was selected as the model condition.

### ICT inhibits oxidative damage and improves apoptosis

**Fig. 3:**
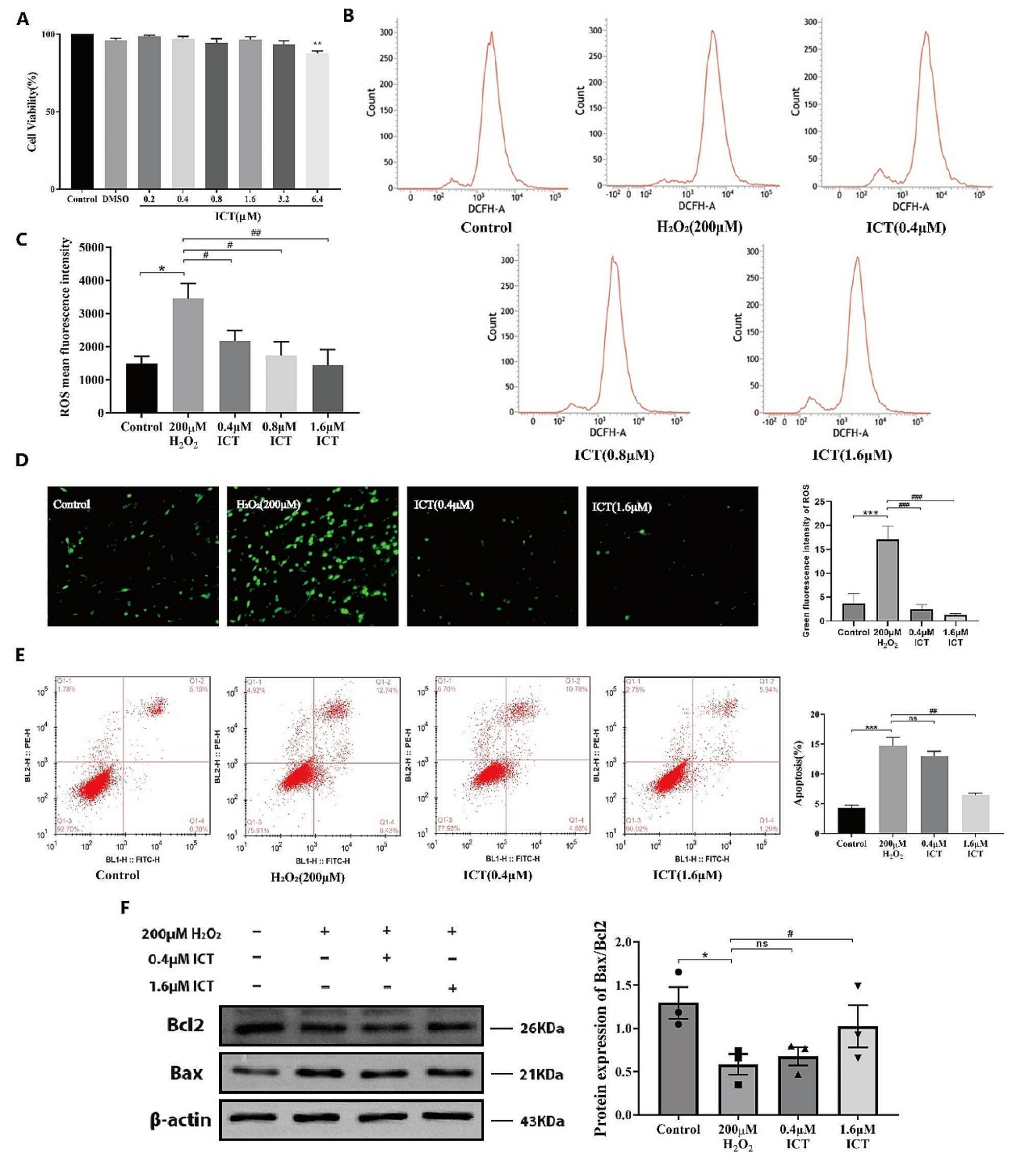
ICT inhibits oxidative damage and apoptosis indcuced by H_2_O_2_. MTT assay measured the protective effect of different concentrations of ICT on H_2_O_2_ treated cell (**A**). Flow cytometry was employed to determine the optimal ICT doseage based on change of ROS levels (**B**-**C**). Immunofluorescence of ROS in each group was utilized to validate of the antioxidant effect of ICT(200×, Scale bar, 100 μm). (**D**). Flow cytometry was used to assess apoptosis levels (**E**). The expression of apoptosis-related proteins Bcl2 and Bax was visualized by Western Blot (**F**). (Data were represented as mean ± SD, *n* = 3, *P < 0.05 vs. control group, **P < 0.01 vs. control group, ***P < 0.001 vs. control group; #*P* < 0.05 vs. model group, ##*P* < 0.01 vs. model group, ##*P* < 0.001 vs. model group)

To explore the protective effect of ICT against oxidative damage, PC-12 cells were pre-treated with various concentrations of ICT 12 h before H_2_O_2_ stimulation. The results showed that ICT effectively inhibit H_2_O_2_-induced cell death within the concentration range of 0.2–3.2 µM (Fig. 3A). Flow cytometry detection of ROS levels showed that under the action of 1.6 µM ICT, the ROS value was close to the normal control, so we chose 0.4 µM and 1.6 µM ICT for subsequent experiments (Fig. 3B-C). The immunofluorescence results showed that ICT treatment effectively suppressed the increase in ROS levels caused by H_2_O_2_ exposure (Fig. 3D). ROS maintains cell survival under normal physiological conditions, but excessive production of ROS can trigger cell apoptosis (Sorokina, et al. 2016; Wang et al. 2023; Papa and Skulachev 1997). The PI staining results showed that the level of cell apoptosis was consistent with the change in ROS level, and ICT effectively inhibited cell apoptosis induced by H_2_O_2_ (Fig. 3E). Furthermore, the expression of the pro-apoptotic protein Bax was notably upregulated following oxidative stress but downregulated with ICT treatment (Fig. 3F). Collectively, these data suggest that ICT reduces ROS levels and apoptosis in the H_2_O_2_-induced oxidative damage model.

### ICT inhibits H_2_O_2_-induced mPTP excessive-opening

**Fig. 4:**
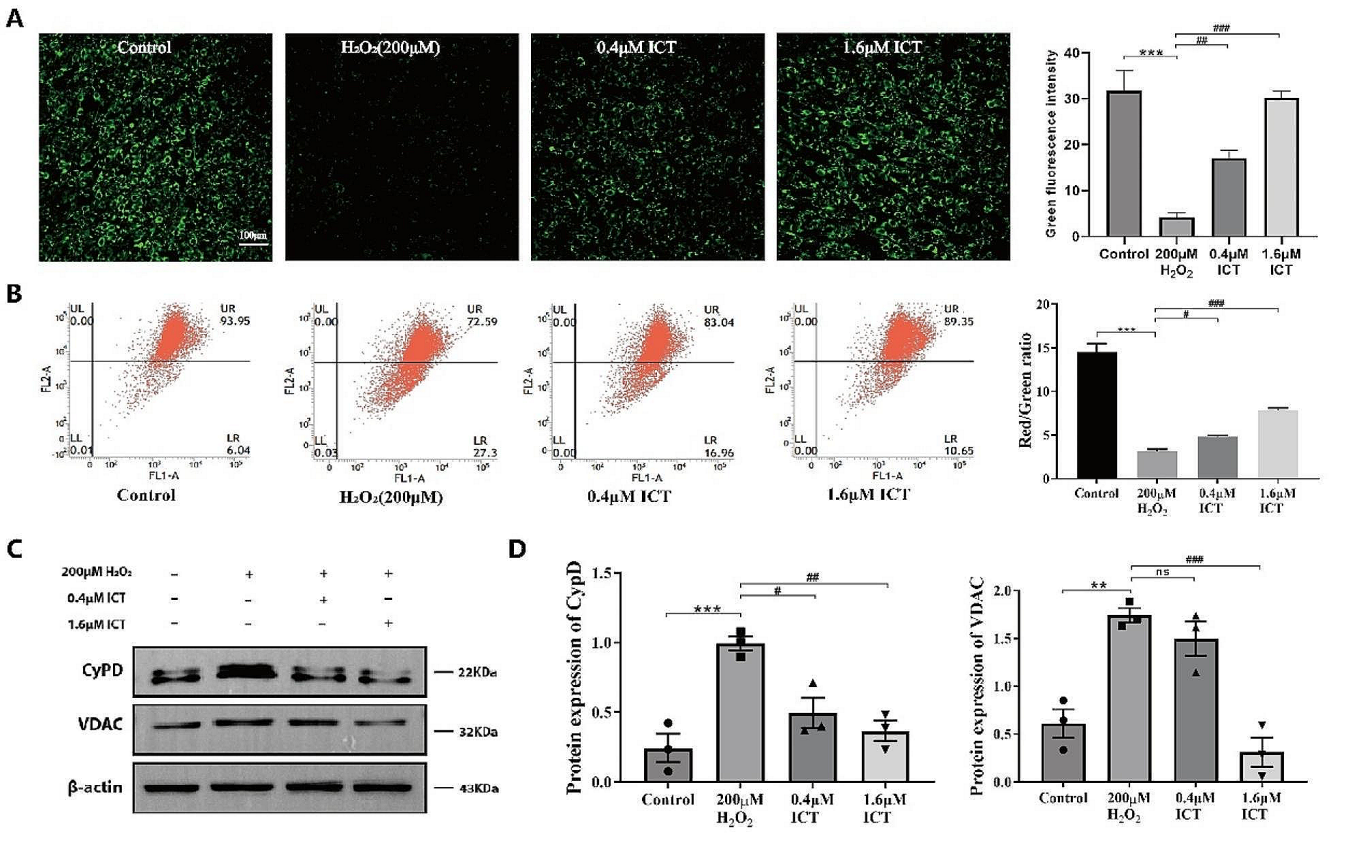
ICT inhibits the excessive opening of mPTP. The effect of ICT on mPTP opening was assessed by calcein staining, where strong fluorescence indicates a closed state and weak fluorescence indicates an open state (200×, Scale bar, 100 μm) (**A**). Flow cytometry was employed to detect change in MMP after ICT administration (**B**). Western Blot was utilized to visualize the expression levels of mPTP related proteins (**C-D**). (Data were represented as mean ± SD, *n* = 3, **P < 0.01 vs. control group, ***P < 0.001 vs. control group; #*P* < 0.05 vs. model group, ##*P* < 0.01 vs. model group, ###*P* < 0.001 vs. model group)

Mitochondria are the major sub-cellular organelles for ROS production. Excessive ROS triggers the opening of mPTP, leading to mitochondrial depolarization and subsequent dysfunction. The calcein assay showed abnormal mPTP opening following H_2_O_2_ treatment, evidenced by a significant decrease in intracellular green fluorescence, which was attenuated by ICT treatment (Fig. 4A). Cerebral ischemia results in a deficiency of O_2_ and glucose, subsequently leading to ATP depletion. This depletion causes the deactivation of ion pumps dependent on mitochondrial membranes, ultimately resulting in a decrease in MMP. Furthermore, alterations in MMP can similarly reflect the levels of mPTP opening. Employing the JC-1 assay, we observed a decrease in MMP levels upon exposure to H_2_O_2_, but these levels reverted to baseline following ICT treatment (Fig. 4B). Furthermore, the expression of Cypd and VDAC (the constituent proteins of mPTP) was upregulated in H_2_O_2_ treatment group, whereas it was suppressed after ICT treatment (Fig. 4C-D). These results remind us that ICT may restore mitochondrial homeostasis by inhibiting H_2_O_2_-induced mPTP excessive opening.

### ICA alleviates MCAO-induced I/R injury in rats

**Fig. 5:**
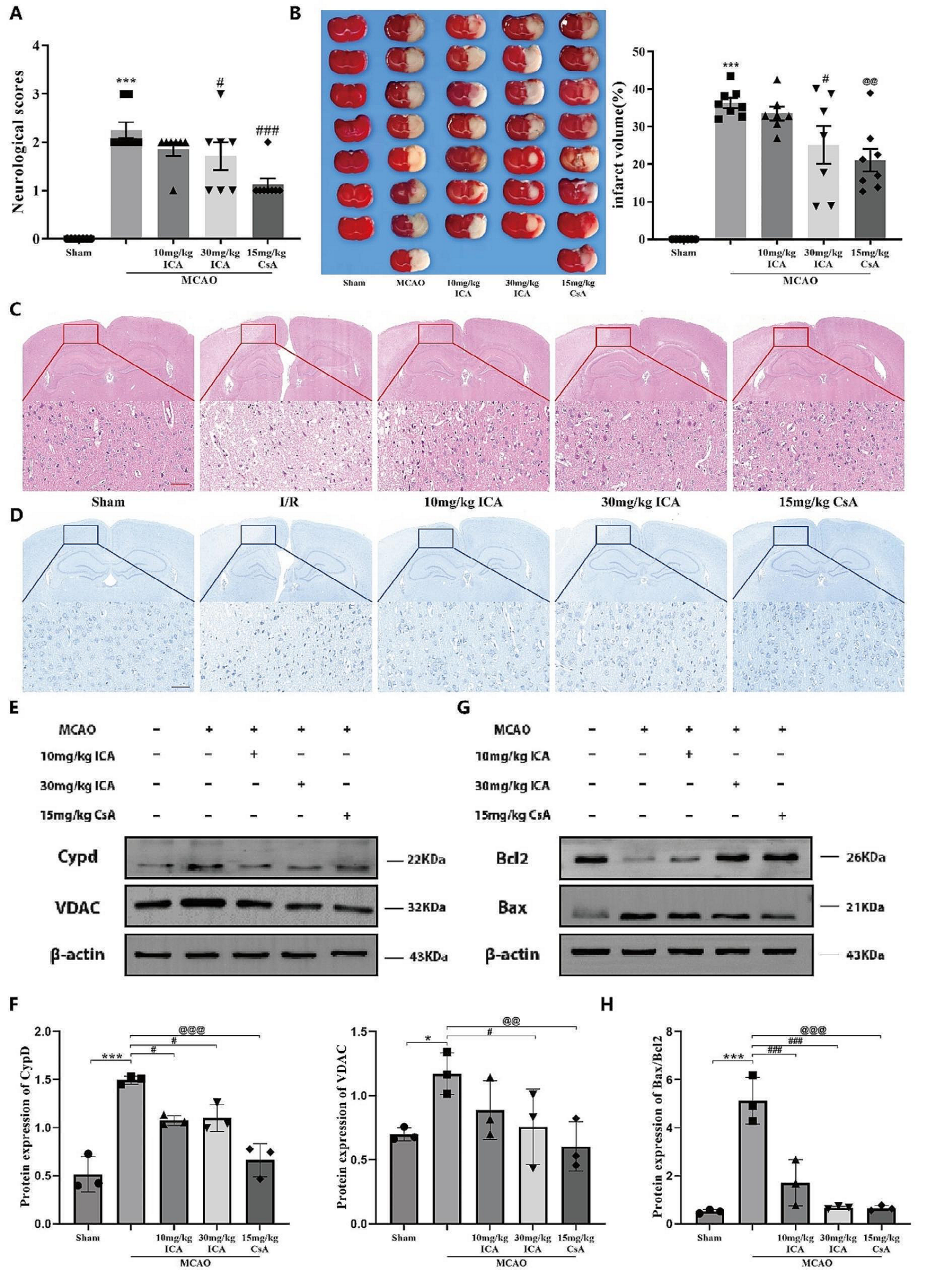
ICA alleviates MCAO-induced I/R injury in rats. Evaluation of ZeaLonga scores following MCAO (**A**). Representative images of TTC-stained coronal brain sections and quantitative analysis of brain infarct volumes (**B**). Representative images of H&E (**C**) and Nissl staining staining **(D)** of the ischemic cortex(40×, Scale bar, 100 μm). Assessment of changes in mPTP (**E**-**F**) and apoptosis-related protein levels (**G**-**H**). (Data were represented as mean ± SD, Neurobehavioural scores and TTC staining in sham-operated group *n* = 8, MCAO group, *n* = 7; 10 mg/kg ICA group, *n* = 7; 30 mg/kg ICA group, *n* = 7; 15 mg/kg CsA, *n* = 7; other results in each group *n* = 3. *P < 0.01 vs. sham-operated group, ***P < 0.001 vs. sham-operated group; #*P* < 0.05 vs. I/R group, ##*P* < 0.01 vs. I/R group, ###*P* < 0.001 vs. I/R group; @@*P* < 0.001 vs. I/R group)

We further evaluated the protective effects of ICA and mPTP specific inhibitor CsA in vivo using MCAO rats. The ZeaLonga scores was evaluated the neurological- function of rats. As shown in Fig. 5A, ZeaLonga scores of MCAO rats exceeded 2, indicating severe disruption of neurological function. Administration of either ICA or CsA individually significantly decreased the ZeaLonga scores, indicating effective restoration of neurological function levels following MCAO-induced I/R injury. Through TTC staining, we can more intuitively observe the changes in cortical infarct volume in each group of rats. As expected, the infarct volume in MCAO rats was significant, while the ICA treatment group showed a dose-dependent decrease (Fig. 5B). The H&E and Nissl staining results showed that the neural cells in the sham surgery exhibited normal morphological structures with clear cytoplasm and nucleus, and orderly arrangement of Nissl bodies. The MCAO group exhibited a sieve-like appearance, vacuolization, and blurred Nissl bodies. However, in the ICA and CsA treatment groups, neuronal morphology recovered and showed a relatively orderly arrangement, with Nissl bodies becoming clearer (Fig. 5C-D). Furthermore, the levels of mPTP-related proteins were upregulated after I/R injury but decreased following ICA or CsA treatment (Fig. 5E-F), indicating that ICA and CsA improve cerebral I/R injury by inhibiting the excessive opening of mPTP in MCAO rats. Continuous mPTP opening has been shown to stimulate neuronal apoptosis. In order to further detect the apoptosis of cells in the infarcted area, we tested the expression levels of Bax and Bcl2 proteins. As shown in Fig. 5G-H, the expression of the pro-apoptotic protein Bax was upregulated following MCAO, whereas the expression of the anti-apoptotic protein Bcl2 was deregulated. As anticipated, the administration of ICA or CsA mitigated the apoptosis induced by MCAO.

### mPTP activation reverses the protective effect of ICT

**Fig. 6:**
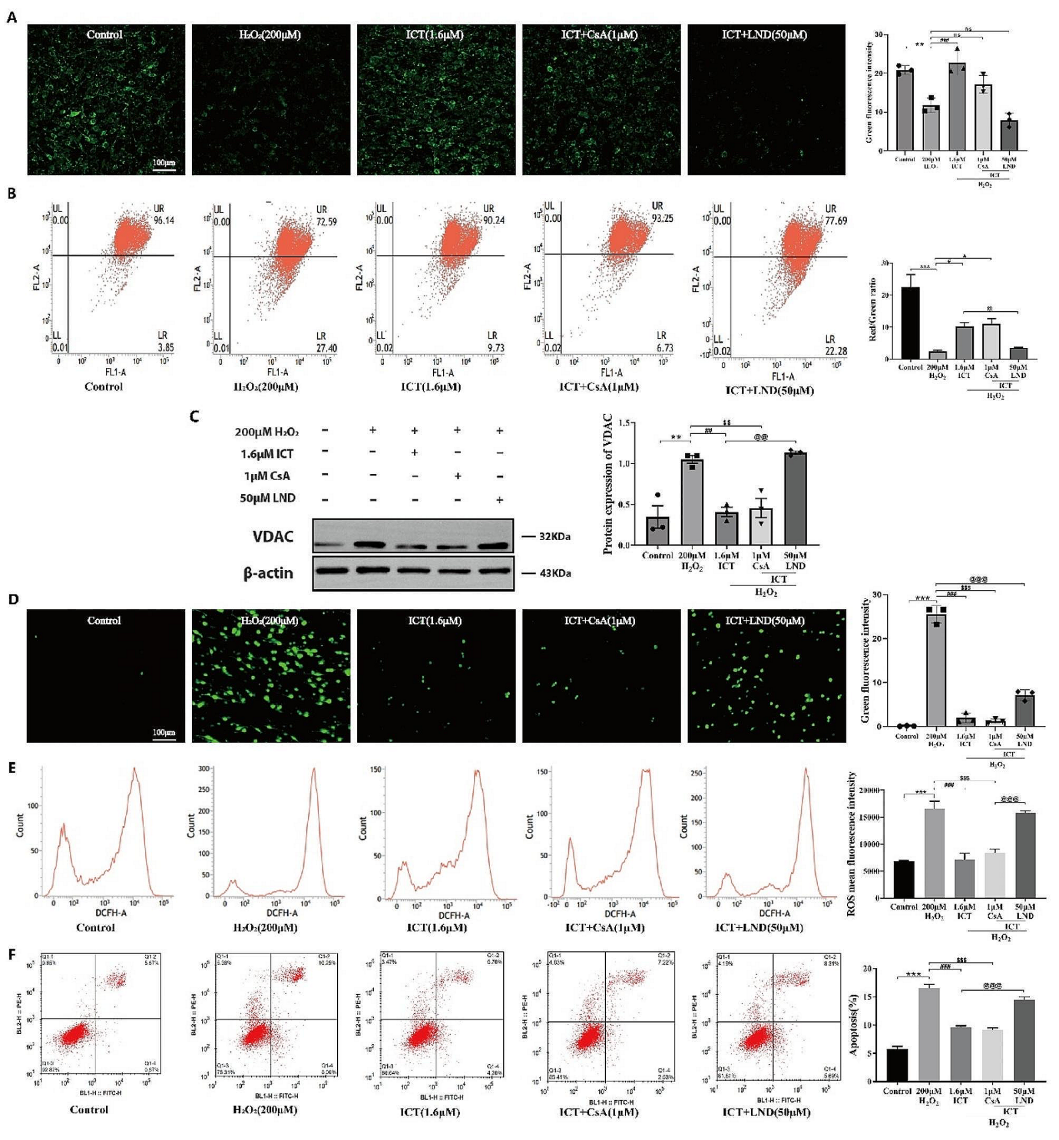
The activation of mPTP reverses the protective effect of ICT. Representative images of immunofluorescence results showing mPTP opening in each group of cells(200×, Scale bar, 100 μm) (**A**). Detection of MMP levels in each group through JC-1 assay (**B**). Analysis of VDAC protein expression and corresponding statistical plots (**C**). Representative images of Immunofluorescence(200×, Scale bar, 100 μm) (**D**) and flow cytometry (**E**) ROS levels across different groups. Assessment of apoptosis in different groups of cells along with statistical plots (**F**). (Data were represented as mean ± SD, *n* = 3, **P < 0.01 vs. control group, ***P < 0.001 vs. control group; #*P* < 0.05 vs. model group, ##*P* < 0.01 vs. model group, ###*P* < 0.001 vs. model group; $$*P* < 0.01 vs. model group, $$*P* < 0.001 vs. model group; @*P* < 0.05 vs. ICT group, @@*P* < 0.01 vs. ICT group, @@@*P* < 0.001 vs. ICT group)

Although ICT has been shown to improve H_2_O_2_-induced oxidative damage and apoptosis in PC-12 cells, it is still unclear whether the protective effect is mediated by mPTP. To further investigate this, we utilized mPTP inhibitor CsA and activator LND to confirm whether ICT exerts protective effects by regulating mPTP opening. Evaluation of mPTP opening using calcein and JC-1 assays revealed that ICT treatment alone significantly inhibited the elevated levels of mPTP and MMP induced by oxidative damage (Fig. 6A-B). Immunoblotting analysis of expression of VDAC yielded consistent results (Fig. 6C). Furthermore, ICT markedly reduced the elevated levels of ROS (Fig. 6D-E) as well as apoptosis (Fig. 6F) induced by H_2_O_2_. Interestingly, these effects were not enhanced by co-treatment with the mPTP inhibitor CsA but were inhibited by co-treatment with the mPTP activator LND (Fig. 6A-E). These results confirm that the antioxidant effects of ICT in I/R injury are mediated by inhibiting mPTP opening, and that activation of mPTP reverses the protective effects of ICT.

## Discussion

Ischemic stroke accounts for 80–85% of strokes, which is twice the lifetime risk of hemorrhagic stroke and lacks effective clinical treatment strategies (Feigin et al. 2016; Johnson et al. 2016; Albers et al. 2018). Therefore, it is urgent to find new targets, develop effective therapeutic drugs to alleviate the burden of stroke. In this study, we confirmed the neuroprotective effect of ICA in the MCAO model in vivo. Moreover, in the H_2_O_2_ induced neuronal oxidative damage in vitro model, ICT can effectively reduce ROS levels as well as inhibit the excessive opening of mPTP. Furthermore, by combining the inhibitor (CsA) and the agonist (LND) of mPTP, it was confirmed that inhibiting the excessive opening of mPTP is a key mechanism for ICT to against I/R induced oxidative damage.

After ischemic stroke, cerebral blood flow immediately decreases, which limits the availability of glucose and oxygen in neurons, and causes serious damage to neuron and brain tissues(Andrabi et al. 2020). Subsequent blood reperfusion process will lead to massive production of ROS and further expand the damage. A previous study demonstrated that ICA protects cerebral neural cells from I/R injury by lowering ROS production and intracellular calcium concentration(Ning and Gao 2023). Here in, we established an in vitro oxidative damage model by treating PC-12 cells with 200µM H_2_O_2_ for 8 h. After pre-treatment with ICT, the mortality rate of model cells was reduced, while the intracellular ROS level was significantly decreased, indicating that ICT is a potential therapeutic drug for alleviating oxidative damage.

Accumulating evidences have proven that ROS were export from mitochondrial through mPTP (Zorov et al. 2014). In addition, previous reports have confirmed that excessive opening of mPTP is a key hub for I/R oxidative damage (Garbaisz et al. 2014). Zhou et al. demonstrated that ICT can improve colon cancer through the mPTP necrosis pathway(Zhou et al. 2016). Here in, PC-12 cells stimulated with H_2_O_2_ showed lower levels of calcein fluorescence intensity than the normal control group, consistent with Western Blot results showing downregulation of mPTP related proteins CyPD and VDAC. These datas implied that I/R injury induced excessive opening of mPTP in vitro.

Under physiological conditions, mPTP periodically opens, allowing positive ions in the membrane gap to enter the matrix, maintaining MMP. After the influence of various stimulating factors, the non-specific opening of mPTP continuously causes positive ions in the membrane gap to enter the matrix, resulting in the disappearance of ion gradients on both sides of the inner membrane and a gradual decrease in MMP (Tiwari et al. 2022). During experiments we also found that oxidative damage seriously decreased MMP. As expected, ICT pre-protection significantly inhibits the opening of mPTP and the increase of MMP induced by I/R injury, manifested by an increase in calcein fluorescence intensity, an increase in jc-1 levels in flow cytometry, and a decrease in mPTP related protein expression (Fig. 6A-D).

The uncoupling of mitochondrial respiratory chain, cessation of ATP synthesis, and efflux of Ca^2+^ lead to excessive opening of mPTP and induce cell apoptosis(Duchen 2004). In addition, the mitochondrial inner membrane is hypertonic, and the excessive opening of mPTP leads to mitochondrial matrix expansion, which is prone to rupture and releases pro apoptotic proteins such as bcl2, ultimately causing cell apoptosis(Reed et al. 1996). Research has shown that ICT counteracts hippocampal neuronal apoptosis through the GR/BDNF signaling pathway(Tang et al. 2020). Wu et al. revealed the therapeutic effect of ICT on Cerebral I/R-induced apoptosis in an acute ischemic stroke mouse model (Wu et al. 2022). In this study, apoptosis levels were detected by the flow cytometry and immunoblotting. Our results are consistent with previous research, I/R injury remarkably increased the number of apoptotic cells, upregulated Bcl2 proteins and decreased the expression of Bax. ICT effectively improves neuronal cell apoptosis caused by I/R injury. Similarly, we also demonstrated that ICA can improve I/R injury in MCAO rat model in vivo, significantly reducing neurological scores and cerebral infarction volume, and improving neuronal morphology. ICA and CsA had similar effects in vivo, both of which can downregulate the protein levels that make up mPTP and inhibit cell apoptosis in brain tissue.

Furthermore, the combined use of mPTP inhibitor CsA and activator LND confirmed that inhibiting excessive opening of mPTP is the key factor for ICT to exert neuroprotective effects. The combined use of inhibitor CsA showed no further protective effect, while the combined use of activator LND reversed the protective effect of ICT. In all, our results suggest that ICA improves I/R injury induced apoptosis by inhibiting mPTP excessive opening. More interestingly, previous studies have also shown that inhibiting the opening of mPTP can improve I/R injury. For example, Gynostemma pentaphyllum extract could protect OGD/R-induced rats isolated hippocampal slices damage by inhibiting neuronal Ca^2+^ overload and mitochondrial oxidative stress-induced mPTP opening (Schild et al. 2009). Gallic acid inhibits the binding of CypD and adenine nucleotide translocators, resulting in a desensitization to induction of mPTP, protecting mitochondria, and ultimately exerting anti I/R effects (Sun, et al. 2017).

The reperfusion period, characterized by the significant production of ROS, is a crucial pathological stage in ischemic stroke, frequently with oxidative stress injury that ultimately culminates in mitochondrial dysfunction and neural injury. Currently, neuroprotective and neurorestorative therapy are two prominent drug intervention strategies for managing ischemic stroke. Our data demonstrated that ICA exhibits remarkable antioxidant activity and exhibits significant effects in enhancing the neural function of MCAO rats, indicating it is a promising candidate for combination therapy with traditional thrombolytic agents like rt-PA. This combined approach aims to mitigate oxidative stress during reperfusion, offer neuroprotective support to stroke patients, and enhance treatment outcomes. Furthermore, ICA holds potential as a neuroprotective agent for post-surgical patients, playing a vital role in prognostic improvement.

There are some limitations in the current study. First, we have found that ICA can inhibit the opening of mPTP, but the upstream regulation pathway is still unclear. Second, ICT as the activity metabolic production of ICA, numerous previously reports have focused on its anti-inflammatory (Lai et al. 2013) and anti-tumor properties (Dongye et al. 2022). Here, we found that ICT has obvious antioxidant effects in vitro, consistent with previous reports (Xu et al. 2021b). However, further confirmation is needed on the efficacy results of direct administration of ICT in vivo. Next, the neuroprotective effect and mechanism of ICT in MCAO rats will be investigated.

## Conclusion

Our study found that ICA can significantly improve I/R injury in vivo, suggesting that ICA can serve as a potential therapeutic drug for ischemic stroke. Furthermore, we found that the protective effect of ICA and its main metabolite ICT in vitro and in vivo is mediated by inhibiting the excessive opening of mPTP, indicating that the inhibitor of mPTP is expected to become a new treatment for ischemic stroke.

## Data Availability

The datasets generated and/or analyzed during the current study are available from the corresponding author on reasonable request.

## Abbreviations

ICA: Icarrin
ICT: Icaritin
I/R: Ischemia-reperfusion
mPTP: Mitochondrial Permeability Transition Pore
MCAO: Middle Cerebral Artery Occlusion
CsA: Cyclosporin A
LND: Lonidamine
ROS: Reactive Oxygen Species
VDAC: Voltage-dependent Anion Channel
ANT: Adenine Nucleotide Translocase
CYPD: Cyclophilin
TTC: 2,3,5-Triphenyltetrazolium Chloride
MTT: Thiazolyl Blue Tetrazolium Bromide
DMSO: Dimethyl Sulfoxide
PBS: Phosphate Buffer Saline
CCA: Common Carotid Artery
ECA: External Carotid Artery
MCA: Middle Cerebral Artery
SDS-PAGE: Polyvinylidene Difluoride
PVDF: Polyvinylidene Difluoride
ECL: Enhanced Chemiluminescence
OGD/R: Oxygen Glucose Deprivation/Re-oxygenation

## References

[CR42] Albers GW, Marks MP (2018). Thrombectomy for Stroke at 6 to 16 hours with selection by Perfusion Imaging. N Engl J Med.

[CR4] Allen CL (2009). Bayraktutan oxidative stress and its role in the pathogenesis of ischaemic stroke. Int J Stroke.

[CR31] An H, Zhou B (2021). Mitochondrial quality control in acute ischemic stroke. J Cereb Blood Flow Metab.

[CR43] Andrabi SS, Parvez S (2020). Ischemic stroke and mitochondria: mechanisms and targets. Protoplasma.

[CR19] Chanoit G, Zhou J (2011). Inhibition of Phosphodiesterases leads to Prevention of the mitochondrial permeability transition pore opening and Reperfusion Injury in Cardiac H9c2 cells. Cardiovasc Drugs Ther.

[CR15] Chaudhuri A, Datta DC, Choi (2016). MicroRNA-7 regulates the function of mitochondrial permeability transition pore by targeting VDAC1 expression. J Biol Chem.

[CR11] Chen HS, Chen X (2018). Potential molecular targets of peroxynitrite in mediating blood-brain barrier damage and haemorrhagic transformation in acute ischaemic stroke with delayed tissue plasminogen activator treatment. Free Radic Res.

[CR8] Dasuri K, Zhang L et al. Oxidative stress, neurodegeneration, and the balance of protein degradation and protein synthesis. Free Radic Biol Med 2013:170–85. 10.1016/j.freeradbiomed.2012.09.016. 10.1016/j.freeradbiomed.2012.09.016 23000246

[CR54] Dongye Z, Wu X, etal, et al. Icaritin and intratumoral injection of CpG treatment synergistically promote T cell infiltration and antitumor immune response in mice. Int Immunopharmacol. 2022;109093. 10.1016/j.intimp.2022.109093. 10.1016/j.intimp.2022.109093 35930912

[CR48] Duchen MR (2004). Mitochondria in health and disease: perspectives on a new mitochondrial biology. Mol Aspects Med.

[CR34] Fan J, Miao Y (2023). Icaritin inhibits oxidative stress in murine astrocytes by binding to Orai1 to block store-operated calcium channel. Chem Biol Drug Des.

[CR40] Feigin VL, Roth GA (2016). Global burden of stroke and risk factors in 188 countries, during 1990–2013: a systematic analysis for the global burden of Disease Study 2013. Lancet Neurol.

[CR45] Garbaisz D, Turoczi Z (2014). Attenuation of skeletal muscle and renal injury to the lower limb following ischemia-reperfusion using mPTP inhibitor NIM-811. PLoS ONE.

[CR6] Groeger G, Quiney C (2009). Hydrogen peroxide as a cell-survival signaling molecule. Antioxid Redox Signal.

[CR17] Hausenloy D, Wynne A (2004). Transient mitochondrial permeability transition pore opening mediates preconditioning-induced protection. Circulation.

[CR41] Johnson W, Onuma O (2016). Stroke: a global response is needed. Bull World Health Organ.

[CR13] Katsu M, Niizuma K (2010). Hemoglobin-induced oxidative stress contributes to matrix metalloproteinase activation and blood-brain barrier dysfunction < i > in vivo. J Cereb Blood Flow Metab.

[CR21] Kinnally KW, Pablo M, Peixoto (2011). Is mPTP the gatekeeper for necrosis, apoptosis, or both?. Biochim Et Biophys Acta-Molecular Cell Res.

[CR53] Lai XQ, Ye YX (2013). Icaritin exhibits anti-inflammatory effects in the mouse peritoneal macrophages and peritonitis model. Int Immunopharmacol.

[CR29] Li Y (2019). Effects of icariside II on brain tissue oxidative stress and Nrf2/HO-1 expression in rats with cerebral ischemia-reperfusion injury1. Acta Cir Bras.

[CR23] Li Y, Sun J et al. Mitochondrial MPTP: a Novel Target of Ethnomedicine for Stroke treatment by apoptosis inhibition. Front Pharmacol 2020:352. 10.3389/fphar.2020.00352. 10.3389/fphar.2020.00352 PMC7109312 32269527

[CR24] Liu D, Ji Q (2022). Cyclosporine A loaded brain targeting nanoparticle to treat cerebral ischemia/reperfusion injury in mice. J Nanobiotechnol.

[CR36] Longa EZ, Weinstein PR (1989). Reversible middle cerebral artery occlusion without craniectomy in rats. Stroke.

[CR35] Ma R, Xie Q (2020). Animal models of cerebral ischemia: a review. Biomed Pharmacother.

[CR22] Nazareth W, Yafei N (1991). Inhibition of anoxia-induced injury in heart myocytes by cyclosporin A. J Mol Cell Cardiol.

[CR20] Neumar RW (2000). Molecular mechanisms of ischemic neuronal injury. Ann Emerg Med.

[CR44] Ning K (2023). Gao Icariin protects cerebral neural cells from ischemia–reperfusion injury in an in vitro model by lowering ROS production and intracellular calcium concentration. Exp Ther Med.

[CR39] Papa S. and V. P. Skulachev. Reactive oxygen species, mitochondria, apoptosis and aging. Mol Cell Biochem 1997; 1–2:305–19. 9309704

[CR7] Patten DA, Germain M, et al. Reactive oxygen species: Stuck in the Middle of Neurodegeneration. J Alzheimers Disease. 2010;S357–67. 10.3233/jad-2010-100498. 10.3233/JAD-2010-100498 20421690

[CR12] Qu J, Chen W (2016). The Injury and Therapy of reactive oxygen species in Intracerebral Hemorrhage looking at Mitochondria. Oxidative Med Cell Longev.

[CR5] Ray PD, Huang BW (2012). Reactive oxygen species (ROS) homeostasis and redox regulation in cellular signaling. Cell Signal.

[CR49] Reed JC, Zha H et al. Structure-function analysis of Bcl-2 family proteins. Regulators of programmed cell death. Adv Exp Med Biol 1996:99–112. 8910675

[CR10] Saeed SA, Shad KF (2007). Some new prospects in the understanding of the molecular basis of the pathogenesis of stroke. Exp Brain Res.

[CR51] Schild L, Roth A (2009). Protection of hippocampal slices against hypoxia/hypoglycemia injury by a Gynostemma pentaphyllum extract. Phytomedicine.

[CR16] Skemiene K, Rekuviene E, et al. Comparison of effects of Metformin, Phenformin, and inhibitors of mitochondrial complex I on mitochondrial permeability transition and ischemic brain Injury. Biomolecules. 2020;10. 10.3390/biom10101400. 10.3390/biom10101400 PMC7600544 33019635

[CR27] Song YH, Cai H, et al. Icariin attenuated oxidative stress induced-cardiac apoptosis by mitochondria protection and ERK activation. Biomed Pharmacother. 2016;1089–94. 10.1016/j.biopha.2016.08.016. 10.1016/j.biopha.2016.08.016 27551754

[CR37] Sorokina, I. V., T. V. Denisenko, et al. Reactive oxygen species regulate a balance between mitotic catastrophe and apoptosis. Int J Biochem Cell Biol. 2016; Pt A:133–136. 10.1016/j.biocel.2016.11.006. 10.1016/j.biocel.2016.11.006 27840153

[CR52] Sun J, Ren DD et al. Desensitizing mitochondrial permeability transition by ERK-Cyclophilin D Axis contributes to the neuroprotective effect of gallic acid against Cerebral Ischemia/Reperfusion Injury. Front Pharmacol 2017:184. 10.3389/fphar.2017.00184. 10.3389/fphar.2017.00184 PMC5382198 28428752

[CR50] Tang C, Liu X (2020). Antagonizing effect of icaritin on apoptosis and injury of hippocampal neurocytes induced by amyloid beta via GR/BDNF signaling pathway. J Recept Signal Transduct Res.

[CR28] Tao H, Liu M et al. Icaritin induces Anti-tumor Immune responses in Hepatocellular Carcinoma by inhibiting Splenic myeloid-derived suppressor cell generation. Front Immunol 2021:609295. 10.3389/fimmu.2021.609295. 10.3389/fimmu.2021.609295 PMC7952329 33717093

[CR47] Tiwari S, Dewry RK, et al. Targeted antioxidant delivery modulates mitochondrial functions, ameliorates oxidative stress and preserve sperm quality during cryopreservation. Theriogenology. 2022;22–31. 10.1016/j.theriogenology.2021.11.013. 10.1016/j.theriogenology.2021.11.013 34823058

[CR3] Tsao CW, Aday AW (2023). Heart Disease and Stroke Statistics-2023 update: a Report from the American Heart Association. Circulation.

[CR9] Turrens JF (2003). Mitochondrial formation of reactive oxygen species. J Physiol.

[CR2] Virani SS, Alonso A (2020). Heart Disease and Stroke Statistics-2020 update: a Report from the American Heart Association. Circulation.

[CR38] Wang BQ, Wang Y (2023). ROS-induced lipid peroxidation modulates cell death outcome: mechanisms behind apoptosis, autophagy, and ferroptosis. Arch Toxicol.

[CR32] Wu CT, Chen MC, et al. Bioactive flavonoids Icaritin and Icariin protect against Cerebral Ischemia-Reperfusion-Associated apoptosis and Extracellular Matrix Accumulation in an ischemic stroke mouse model. Biomedicines. 2021;11. 10.3390/biomedicines9111719. 10.3390/biomedicines9111719 PMC8615444 34829948

[CR33] Wu CT, Yang TH, et al. Therapeutic effect of Icaritin on Cerebral Ischemia-Reperfusion-Induced Senescence and apoptosis in an Acute Ischemic Stroke Mouse Model. Molecules. 2022;18. 10.3390/molecules27185783. 10.3390/molecules27185783 PMC9500895 36144517

[CR1] Xie W, Zhu T (2020). Notoginseng Leaf Triterpenes ameliorates OGD/R-Induced neuronal Injury via SIRT1/2/3-Foxo3a-MnSOD/PGC-1α signaling pathways mediated by the NAMPT-NAD pathway. Oxidative Med Cell Longev.

[CR26] Xu Y, Lu X (2021). Icaritin activates Nrf2/Keap1 signaling to protect neuronal cells from oxidative stress. Chem Biol Drug Des.

[CR55] Xu YY, Lu XY (2021). Icaritin activates Nrf2/Keap1 signaling to protect neuronal cells from oxidative stress. Chem Biol Drug Des.

[CR14] Yunoki T, Deguchi K et al. Anti-oxidative nutrient rich diet protects against acute ischemic brain damage in rats. Brain Res 2014:33–9. 10.1016/j.brainres.2014.08.056. 10.1016/j.brainres.2014.08.056 25175837

[CR46] Zhou C, Chen Z (2016). Icaritin activates JNK-dependent mPTP necrosis pathway in colorectal cancer cells. Tumour Biol.

[CR25] Zhou X, Wang HY (2017). Ginkgolide K attenuates neuronal injury after ischemic stroke by inhibiting mitochondrial fission and GSK-3β-dependent increases in mitochondrial membrane permeability. Oncotarget.

[CR30] Zhu HR, Wang ZY (2010). Icariin protects against brain injury by enhancing SIRT1-dependent PGC-1alpha expression in experimental stroke. Neuropharmacology.

[CR18] Zorov DB, Juhaszova M (2014). Mitochondrial reactive oxygen species (ROS) and ROS-induced ROS release. Physiol Rev.

